# A comparison of the different anthropometric indices for assessing malnutrition among older people in Turkey: a large population-based screening

**DOI:** 10.1186/s41043-021-00228-z

**Published:** 2021-03-30

**Authors:** Gülüşan Özgün Başıbüyük, Parvin Ayremlou, Sakineh Nouri Saeidlou, Faruk Ay, Akgül Dalkıran, Wida Simzari, Gábor Áron Vitályos, Yener Bektaş

**Affiliations:** 1 Department of Gerontology, Faculty of Health Sciences, Akdeniz University, Antalya, Turkey; 2 Clinical Research Development Unit of Imam Khomeini Hospital, Urmia University of Medical Sciences, Urmia, Iran; 3 Food and Beverages Safety Research Center, Urmia University of Medical Sciences, Urmia, Iran; 4 Department of Anthropology, Faculty of Literature, Sivas Cumhuriyet University, Sivas, Turkey; 5 Department of Nutrition and Dietetic, School of Health Sciences, Coppadocia University, Nevşehir, Turkey; 6 Department of Natural Sciences, Faculty of Primary and Pre-School Education, Eötvös Loránd University, Budapest, Hungary; 7 Department of Archaeology, Faculty of Arts and Sciences, Nevşehir Hacı Bektaş Veli University, Nevşehir, Turkey

**Keywords:** Nutritional status, Anthropometry, Malnutrition, Older people

## Abstract

**Objective:**

Due to an increase in aging worldwide, assessment of the nutritional status of older people becomes an important matter. Malnutrition in older people increases the risk of infections, disease period and hospitalization rates. This study aimed to compare the different anthropometric indices for detecting malnutrition among older people and comparing these indices among males and females to explain the possible differences.

**Methods:**

In this cross-sectional study, 2721 aged 65 years and older in Turkey were enrolled. Anthropometric measurements weight, height, hip circumference (HC), and waist circumference (WC), abdominal circumference (AC), mid-upper arm circumference (MUAC), triceps skinfold thickness (TST), calf circumference (CC)) were measured. Body mass index (BMI), abdominal volume index (AVI), body roundness index (BRI) and body adiposity index (BAI), and waist-to-hip ratio (WHR) and waist-to-height ratio (WHtR) indices were calculated using standardized formulas. The receiver operator characteristic curves (ROCs) were conducted in detecting the best anthropometric parameters. Adjusted odds ratios (OR) (stratified by sex) calculated for each anthropometric index.

**Results:**

Participants with both BMI < 18.5 (1.1%) and BMI > 25 (80%) defined as the malnourished group and BMI of 18.5–24.99 (18.9%) defined as the normal group. In both sexes, the area under the curve (AUC) was > 0.7 for all anthropometric indices except WHR in females (AUC 0.66). BRI, WHR, WHtR, and AVI indices strongly predict the risk of malnutrition among both sexes. In males, the ORs were for BRI (6.83, 95% CI 5.39–8.66), WHR (6.43, 95% CI 5.9–6.9), AVI (2.02, 95% CI 1.86–2.12). In females, the ORs were for BRI (3.72, 95% CI 3.09–4.48), WtHR (2.63, 95% CI 1.3–3.5), and WHR (2.45, 95% CI 1.9–3.06).

**Discussion:**

The presence of a large AUC in almost all anthropometric indices suggests that they can be used to assess the risk of malnutrition among older persons in both sexes.

## Introduction

Aging is increasing worldwide. The number of people aged 65 or over is projected to grow from an estimated 524 million in 2010 to nearly 1.5 billion in 2050, with most of the increase in developing countries in recent years [1, 2]. Physiological changes such as a decreased sense of taste or smell, or both in older people can related with adversely nutritional status. Ageing may also be associated with profound psychosocial and environmental changes, such as isolation, loneliness, depression, and inadequate finances, which may also have significant impacts on diet [3].

Nutritional status of older people is an important factor and it related with quality of life among them [4]. Malnutrition is common condition in older persons and it affects almost 13–78% of the older population [5, 6]. Malnutrition is as an important factor for predicting morbidity and mortality among older persons [4]. Poor nutritional status in older people increases the risk of infections, disease period, poor wound healing, hospitalization rate, postoperative complications [7–11].

Nutritional status of the older people has been evaluated using various tools and methods in different studies [12–21]. An available screening and assessment methods for malnutrition is anthropometric measurements [22–24]. Common anthropometric indices of body composition such as body mass index (BMI), waist circumference (WC), waist-to-hip ratio (WHR), waist-to height ratio (WHtR), arm circumference (AC) and calf circumference (CC) have been widely used [5, 17, 25, 26]. The anthropometric indices are simple, easily obtainable, inexpensive, noninvasive measures of assessing and early detection of malnutrition in older people [22, 26]. Among them BMI is a valid and accepted anthropometric measure indicate underweight, overweight and obesity as all forms of malnutrition [23, 27]. For adults with 20 years of age and older, BMI categories ranges are underweight (BMI<18.5 kg/m2), normal weight (18.5 to <24 kg/m2), overweight (24 to < 28 kg/m2), and obesity (≥28 kg/m2) [28]. Recently, new anthropometric indices including body roundness index (BRI), body adiposity index (BAI), abdominal volume index (AVI), and body shape index (ABSI) have been considered as a predictor of health status [27, 29–31]. Association between these new indices and some diseases has been evaluated [32–34].

Anthropometric indices are affected by several factors including gender, environmental and socioeconomic status, genetic, race, and other factors [35, 36].

Malnutrition leads to poor outcomes and poor quality of life among older persons; therefore, early detection of malnutrition and subsequent nutritional intervention can significantly benefit vulnerable populations in addition to economic benefits such as reducing medical/healthcare costs. Current study assesses the different anthropometric indices for detecting malnutrition among older people and comparing these indices between males and females to explain the possible differences in a large population-based screening in Turkey.

## Methods

### Study population

In this large population-based cross-sectional study, the anthropometric indices were measured in 2721 persons aged over 65 years from both sexes. Cluster sampling method was conducted for the data collection. First, research population was divided into seven geographical/political region of Turkey including Central Anatolia, Aegean, Mediterranean, Black Sea, Marmara, East Anatolia, and Southeast Anatolia. In these 7 regions, 26 provinces were included in this study. Provinces with high older population size were selected according to the population database of Turkish Statistical Institute (TSI). The percentage of participants to represent each region was estimated from the total of older population of each region based on database of TSI. Finally, participants were included in both rural and urban regions applying simple random sampling. Sample size was determined according to α = 0.01, standard deviation (σ) = 15.17, *d* = 0.75 and number of older persons in the + 65 years (*N* = 3327593) using following formula:

$$ N=\frac{N\;{t}^2\;\sqrt{2}}{\left(\mathrm{N}-1\right)\;{\mathrm{d}}^2+{\mathrm{t}}^2\;\sqrt{2}}=\frac{3327593\times {2.58}^2\times {15.17}^2}{\left(3327593-1\right)\times {0.75}^2+{2.58}^2\times {15.17}^2}=2721 $$

The number of older individuals to be included for each region was calculated as 396, 473, 274, 319, 988, 148, and 134 in Central Anatolia, Aegean, Mediterranean, Black Sea, Marmara, East Anatolia, and Southeast Anatolia, respectively.

The study protocol was approved by the Ethics Committee of Cumhuriyet University, Turkey, with 2014-03/15 number.

### Anthropometry assessments

The anthropometric indices were measured according to the techniques described by the Anthropometric Standardization Reference Manual (ASRM) and the International Biological Program (IBP) [37]. The anthropometric measurements were taken by two trained staffs in each participant. The field study was carried out between December 2016 and August 2017. The measurements were taken only once for the most participants. However, a pilot study was conducted to show the invariance of the variability criterion between individual measurements by measuring 35 older adults three times within a month in Sivas Province. The body weight was measured without shoes and minimum clothing to the nearest 0.01 kg using a digital scale (AR550 Sottile Digital Glass Scale, TR). Height was measured in the standing position without shoes using strip meter with accuracy of 0.1 cm (Harpenden Anthropometer, Holtain Ltd., UK). WC was measured using the tape measure in standing with feet shoulder width apart position in the area between the hip bone and under the navel. HC is measured at the widest part of the hips. The largest circumference between the waist and the knees was considered as HC. AC is measured at the midpoint of the line between the rib or costal margin and the iliac crest in the midaxillary line. Mid-upper arm circumference (MUAC) was taken with tape measure. The participant should stand upright, the arm should be freely hanging to the side, and the biceps should be measured without applying pressure to the circumference of the most bulging (middle of the forearm). CC was measured by wrapping the tape around the widest part of the calf in a sitting position. The participant sits at the table with his feet hanging freely. The person taking the measurement stands in front of the participant and measures the maximum circumference of the calf with a tape measure. For measuring of triceps skinfold thickness (TST), the participant should be freely hanging from side to side without stretching his/her arms and the person taking the measurement should be behind the participant. The measurement is taken over the triceps muscles on the back of the upper arm and in the middle of the upper arm (between the acromion and olecranon points) with the skinfold (Skinfold Caliper, Holtain Ltd., UK). Measurements were taken between these two points with tape measure. BMI, BRI, BAI, AVI, WHtR, and WHR indices were calculated using the following standardized formulas:

1. BMI = weight (kg)/height(m^2^) [38]
2. $$ BRI=364.2-365.5\sqrt{1-\left\{\frac{{\left( WC\left[m\right]/2\pi \right)}^2}{0.5\kern0em height\;\left[m\right]\Big){}^2}\right\}} $$ [39, 40]
3. *BAI* = [*HC*(*cm*)/*Height*^1.5^ (*m*)] [30, 41]
4. *AVI* = [2*WC*^2^(*cm*) + 0.7(*WCC* − *HC*)^2^(*cm*)]/1000 [42]
5. *WHtR* = *WC*(*cm*)/*Height*(*cm*) [43]
6. *WHR* : *WC* (*cm*)/*HC*(*cm*) [42]

### Statistical analysis

Continuous variables were presented as means ± SD and categorical variables were expressed as number and percentage. Based on BMI as common and accepted measure for assessing malnutrition, participants characterized within two groups: participants with BMI < 18.5 (underweight) and BMI > 25 (overweight and obesity) as the malnourished group and participants with BMI of 18.5–24.99 as the normal group [38, 44]. The baseline characteristics were compared between normal and malnourished groups using the Pearson’s chi-square test for categorical variables. The mean of anthropometric variables was compared by independent *t* test between two groups stratified by sex. The area under the receiver operating characteristic curve (AUC) was used to assess the predictive ability of anthropometric measurements for the malnutrition (stratified by sex). The odds ratios (ORs) and their 95% CIs for the risk of malnutrition for each anthropometric index were calculated by logistic regression. Logistic regression model was adjusted for age groups (65–74, 75–84, and > 85 years), marital status (single, married, and widow), and job (employer, worker, self-employment, pensionary, un-employment, and housekeeper) as categorical variables.

## Results

In this cross-sectional study, 2721 aged 65 years and older in Turkey were enrolled. Of 2721 subjects, 513 (18.9%) participant had normal status and 2208 (81.1%) were malnourished. The percent of participants with BMI < 18.5, 18.5–24.99, 25–29.99, and > 30 was 29 (1.1%), 513 (18.9%), 880 (32.3%), and 299 (47.7%), respectively.

The main baseline characteristics of participants were presented in the Table 1. The sex, age groups, and marital status had statistically significant difference between normal and malnourished groups. One thousand five hundred thirty (56.23%) were females and 1193 (43.77%) were males. In the malnutrition group, 60.7% of subjects were females while 39.3% of whom were males. The prevalence of malnutrition was 62%, 31.8%, and 6.2% among 65–74, 75–84, and > 85 years, respectively. In the normal group, the frequency of single, married, and widow participants was 17.7%, 25.5%, and 56.7%, respectively, while these frequencies were 10.4%, 35.4.5%, and 54.2%, respectively, in the malnutrition group. 46.4% of malnourished participants were housekeepers while in the normal group the subjects with pensionary job status (36.3%) had the highest frequency (*p* < 0.05).

**Table 1:** Comparison of the baseline characteristics between normal and malnourished participants

		Nutrition status		
**Variables**		**Normal (*n*** **= 513)**	**Malnourished (*n*** **= 2208)**	***p*** **value**_Trend *_
**Sex,** ***n*** **(%)**	Male	324(63.2)	867(39.3)	< 0.001
	Female	189(36.8)	1341(60.7)	
**Age (year),** ***n*** **(%)**	65–74	287(55.9)	1368(62)	< 0.001
	75–84	168(32.7)	702(31.8)	
	> 85	58(11.3)	138(6.2)	
**Marital status,** ***n*** **(%)**	Single	91(17.7)	229(10.4)	< 0.001
	Married	131(25.5)	782(35.4)	
	Widow	291(56.7)	1197(54.2)	
**Education level,** ***n*** **(%)**	Illiterate	184(35.9)	760(34.4)	0.852
	Primary school	184(35.9)	835(37.8)	
	Middle	42(8.2)	189(8.6)	
	High school	62(12.1)	240(10.9)	
	College	41(8)	184(8.3)	
**Income,** ***n*** **(%)**	0–999 TL	266(51.9)	1128(51.1)	0.194
	1000–1499 TL	114(22.2)	488(22.1)	
	1500–1999 TL	105(20.5)	412(18.7)	
	2000– + TL	28(5.5)	180(8.2)	
**Job,** ***n*** **(%)**	Employer	7(1.4)	14(0.6)	< 0.001
	Worker	6(1.2)	21(1)	
	Self-employment	163(31.8)	368(16.7)	
	Pensionary	186(36.3)	771(34.9)	
	Un-employment	9(1.8)	9(0.4)	
	Housekeeper	142(27.7)	1025(46.4)	
**Region,** ***n*** **(%)**	Central Anatolia	73(14.2)	323(14.6)	0.098
	Mediterranean	52(10.1)	222(10.1)	
	Black Sea	40(7.8)	279(12.6)	
	Marmara	196(38.2)	781(35.4)	
	East Anatolia	30(5.8)	118(5.3)	
	Southeast Anatolia	30(5.8)	104(4.7)	
	Aegean	92(17.9)	381(17.3)	

^*^Data analyzed by person`s chi-square

The mean of anthropometric indices was compared between normal and malnourished older persons (Table 2). All of anthropometric indices were significantly higher in malnourished group than normal group in total participants, men and women. However, most of these indices were higher in women than in men.

**Table 2:** Comparison of the anthropometric indices between normal and malnourished participants

	Total			Men			Women		
Indices	Normal (***n*** = 513)	Malnourished (***n*** = 2208)	***p*** value	Normal (***n*** = 324)	Malnourished (***n*** = 867)	***p*** value	Normal (***n*** = 189)	Malnourished (***n*** = 1341)	***p*** value*
**HC**	88.74 ± 5.98	101.28 ± 10.48	< 0.001	88.98 ± 5.99	99.34 ± 7.56	< 0.001	88.33 ± 5.94	102.54 ± 11.83	< 0.001
**WC**	79.84 ± 7.37	95.56 ± 10.55	< 0.001	81.79 ± 6.62	98.36 ± 9.24	< 0.001	76.50 ± 7.41	93.75 ± 10.95	< 0.001
**WHR**	0.90 ± 0.08	0.95 ± 0.09	< 0.001	0.92 ± 0.06	0.99 ± 0.07	< 0.001	0.86 ± 0.09	0.91 ± 0.10	< 0.001
**WHtR**	0.51 ± 0.05	0.61 ± 0.07	< 0.001	0.50 ± 0.04	0.60 ± 0.05	< 0.001	0.51 ± 0.06	0.63 ± 0.07	< 0.001
**AC**	88.80 ± 6.47	105.35 ± 10.81	< 0.001	88.38 ± 5.99	103.47 ± 9.94	< 0.001	89.52 ± 7.16	106.57 ± 11.17	< 0.001
**MUAC**	21.37 ± 2.33	26.10 ± 3.40	< 0.001	21.64 ± 2.26	25.75 ± 3.03	< 0.001	20.91 ± 2.40	26.33 ± 3.60	< 0.001
**TST**	5.14 ± 5.21	12.08 ± 7.14	< 0.001	3.30 ± 4.28	8.17 ± 5.55	< 0.001	8.30 ± 5.17	14.62 ± 6.91	< 0.001
**CC**	27.60 ± 3.12	31.95 ± 4.11	< 0.001	28.22 ± 2.77	32.63 ± 3.87	< 0.001	26.54 ± 3.40	31.50 ± 4.21	< 0.001
**AVI**	12.96 ± 2.28	18.56 ± 4.09	< 0.001	13.52 ± 2.11	19.53 ± 3.67	< 0.001	11.98 ± 2.23	17.93 ± 4.22	< 0.001
**BRI**	3.54 ± 0.96	5.95 ± 1.77	< 0.001	3.43 ± 0.76	5.48 ± 1.38	< 0.001	3.75 ± 1.19	6.25 ± 1.93	< 0.001
**BAI**	26.98 ± 4.97	34.90 ± 7.78	< 0.001	24.82 ± 3.19	29.27 ± 4.46	< 0.001	30.68 ± 5.30	38.55 ± 7.3	< 0.001

^*^p value for Independent *t* test

*Abbreviations*: *HC* hip circumference, *WC* waist circumference, *WHR* waist-to-hip ratio, *WHtR* waist-to-height ratio, *AC* abdominal circumference, *MUAC* mid-upper arm circumference, *TST* triceps skinfold thickness, *CC* calf circumference, *AVI* abdominal volume index, *BRI* body roundness index, *BAI* body adiposity index

The area under the curve (AUC) and cut off points of anthropometric indices were presented in Table 3. In general, the most significant AUCs were > 0.8 in both males and females for all anthropometric indices except WHR and TSC in males (AUC 0.78) and WHR in females (AUC 0.66). In males, both of WC and AVI had the same largest AUC (0.94, 95% CI = 0.93–0.95), while both of WHR and TSC had the same smallest AUC (0.78, 95% CI = 0.76–0.81). In descending order, AUC was for both WHtR and BRI (0.93, 95% CI = 0.91–0.94), AC (0.92, 95% CI = 0.91–0.93), UAC (0.89, 95% CI = 0.87–0.91), HC (0.86, 95% CI = 0.0.85–0.9), both of BAI and CC (0.82, 95% CI = 0.8–0.85), and both of WHR and TST (0.78, 95% CI = 0.76–0.81), respectively.

**Table 3:** Area under the curve (AUC), optimal cutoff point, sensitivity, and specificity by receiver-operator curves (ROC) analysis of anthropometric indices to predict malnutrition

Sex	Indexes	Sensitivity, %	Specificity, %	Cut off	AUC	95% CI
**Men**	**HC**	80	80	93.15	0.86	0.85–0.9
	**WC**	86	90	89.95	0.94	0.93–0.95
	**WHR**	80	64	0.94	0.78	0.76–0.81
	**WHtR**	89	80	0.55	0.93	0.91–0.94
	**AC**	81	90	95.95	0.92	0.91–0.93
	**MUAC**	79	84	23.95	0.89	0.87–0.91
	**TST**	70	72	5.45	0.78	0.76–0.81
	**CC**	70	82	30.75	0.82	0.8–0.85
	**AVI**	91	81	15.45	0.94	0.93–0.95
	**BRI**	85	86	4.23	0.93	0.91–0.94
	**BAI**	79	68	26.36	0.82	0.8–0.85
**Women**	**HC**	79	84	93.05	0.88	0.86–0.9
	**WC**	82	82	83.75	0.91	0.89–0.93
	**WHR**	66	60	0.87	0.66	0.62–0.7
	**WHtR**	89	70	0.54	0.88	0.86–0.91
	**AC**	80	87	97.95	0.92	0.9–0.93
	**MUAC**	79	86	23.9	0.9	0.88–0.93
	**TST**	74	72	10.45	0.79	0.76–0.83
	**CC**	70	80	29.45	0.82	0.79–0.86
	**AVI**	90	73	13.18	0.91	0.9–0.93
	**BRI**	87	75	4.27	0.88	0.85–0.91
	**BAI**	82	70	32.63	0.84	0.82–0.87

*Abbreviations*: *HC* hip circumference, *WC* waist circumference, *WHR* waist-to-hip ratio, *WHtR* waist-to-height ratio, *AC* abdominal circumference, *MUAC* mid-upper arm circumference, *TST* triceps skinfold thickness, *CC* calf circumference, *AVI* abdominal volume index, *BRI* body roundness index, *BAI* body adiposity index

In females, AC, WC, AVI, and MUAC had the largest AUC (AC 0.92, 95% CI = 0.9–0.93, both of WC and AVI 0.91, 95% CI = 0.9–0.93 and UAC 0.9, 95% CI = 0.088–0.93). WHR had the smallest AUC (0.66, 95% CI = 0.62–0.7). AUC was similar for HC, WHtR, and BRI (0.88, 95% CI = 0.85–0.91). AUC was for BAI (0.84, 95% CI = 0.82-0.87), for CC (0.82, 95% CI = 0.79–0.86), and TST (0.79, 95% CI = 0.76–0.83) in descending order.

Generally, there were gender differences in the cutoff point of all the anthropometric indices with approximately similar in sensitivity and specificity except for HC, WHtR, MUAC, and BRI. The cutoff point for WC was 89.95 cm and 83.75 cm in males and females, respectively. The cutoff points were for WHR, AC, TST, CC, AVI, and BAI 0.94, 95.95, 5.45, 30.75, 15.45, and 26.36 in males, respectively. While the cutoff points of these indices were in females 0.87, 97.95, 10.45, 29.45, 13.18, and 32.63, respectively.

Adjusted ORs of malnutrition risk for each anthropometric index were shown in Table 4. In both sexes, the significant OR of BRI and WHR were greater than other anthropometric indices. As the OR of BRI was OR 6.83, 95% CI 5.39–8.66 for males and OR 3.72, 95% CI 3.09–4.48 for females. For WHtR the OR was OR 6.43, 95% CI 5.9–6.9 in males and OR 2.45, 95% CI 1.9–3.06, *p* < 0.001 in females. In both sexes, the OR was highest following the two above indices for AVI and MUAC (OR “AVI”: 2.02 and 2.01 in males and females, respectively, and OR “MUAC”: 1.96 and 1.81 in males and females, respectively). Other anthropometric indices were positively associated with malnutrition in both sexes.

**Table 4:** Adjusted odds ratios (ORs)^+^ of malnutrition risk* for each anthropometric index

	Men		Women	
Indices	OR(95% CI)	***p*** value	OR(95% CI)	***p*** value
**HC**	1.26(1.23–1.30)	< 0.001	1.6(1.14–1.19)	< 0.001
**WC**	1.28(1.24–1.32)	< 0.001	1.25(1.21–1.29)	< 0.001
**WHR**	6.43 (5.9–6.9)	< 0.001	2.45 (1.9–3.06)	< 0.001
**WHtR**	1.9 (1.1–1.32.5)	< 0.001	2.63 (1.3–3.5)	< 0.001
**AC**	1.28(1.24–1.32)	< 0.001	1.22(1.18–1.25)	< 0.001
**MUAC**	1.96(1.80–2.13)	< 0.001	1.81(1.67–1.96)	< 0.001
**TST**	1.21(1.17–1.24)	< 0.001	1.18(1.15–1.22)	< 0.001
**CC**	1.45(1.38–1.53)	< 0.001	1.38(1.32–1.46)	< 0.001
**AVI**	2.02(1.86–2.12)	< 0.001	2.01(1.82–2.17)	< 0.001
**BRI**	6.83(5.39–8.66)	< 0.001	3.72(3.09–4.48)	< 0.001
**BAI**	1.41(1.34–1.48)	< 0.001	1.24(1.19–1.28)	< 0.001

^**+**^Adjusted model for age, marital status and job

*Malnutrition was defined as BMI < 18.5 and BMI > 25

*Abbreviations***:** *HC* hip circumference, *WC* waist circumference, *WHR* waist-to-hip ratio, *WHtR* waist-to-height ratio, *AC* abdominal circumference, *MUAC* mid-upper arm circumference, *TST* triceps skinfold thickness, *CC* calf circumference, *AVI* abdominal volume index, *BRI* body roundness index, *BAI* body adiposity index

## Discussion

The anthropometric indices are closely related to the nutritional status. The anthropometric assessment in older people is inexpensive, non-invasive and it can reflect the nutritional status of a population [25]. Among the anthropometric indexes, BMI was used commonly for evaluating of malnutrition [23, 27].

In current study participants were categorized into two groups: BMI < 18.5 (underweight) and BMI > 25 (overweight and obesity) as the malnourished group and participants with BMI of 18.5–24.9 as the normal group. The results showed that only 18.9% of participants had the normal status while 81.1% of them were malnourished. Sanchez-Garcia et al. showed 62.3% of Mexican older persons (> 60 years old) had BMI > 25 and 1.4% had BMI < 18.5 [25]. Setiati et al. reported that 45.01% of older people had normal nutritional status based on BMI and 54.99% of them were malnourished in Indonesia [45]. In India, Kalaiselv et al. showed that 37.6 of older adults had normal BMI (BMI 18.5–23 kg/m^2^) [46]. Despite the differences in eating habits around the world, the prevalence of malnutrition in the older population appears to be high in different regions of the world. Therefore, aging and the factors that lead to an increase in malnutrition in the older population should be taken in consideration.

Current study showed that the AUC of all anthropometric indices in detecting malnutrition was greater than 0.7 in both sexes except WHR in females (AUC 0.66). In both sexes, WC and AVI had the largest AUC (> 0.9). Correa et al. showed that the AUC for waist-to-height ratio (WHtR) as an anthropometric indicator of overweight according to the body mass index (BMI) classification was greater than 0.8 [26]. Consistent with our findings in a study by Jamir et al., the AUC for mid-upper-arm circumference (MUAC), triceps skinfold thickness (TST), and calf circumference (CC) was 0.93, 0.88, and 0.86 in men and 0.95, 0.9, and 0.91 in women, respectively [2].

Findings of this study revealed that there are gender differences in the cutoff points of all the anthropometric indices as nearly similar in sensitivity and specificity. The cutoff point of WC (value 93.15), WHR (value 0.94), CC (value 30.75), and AVI (value 15.45) in males was higher than females (WC value 83.75, WHR value 0.87, CC value 10.45, and AVI value 13.8). However, the cutoff point for AC (value 97.95), TST (value 10.45), and BAI (value 32.63) indices in females was higher than males (value 95.95, 5.45, 26.3 for AC, TST, and BAI, respectively). The cutoff point of HC, WHtR, MUAC, and BRI was nearly similar in both sexes. Consistent with current study, the cutoff point values reported 26.6, 7.9, and 30.7 for MUAC, TST, and CC in males and 25.9, 12, and 28.1 in females, respectively [2]. In a study, the cutoff point of WHtR and BRI was similar in both sexes (0.51 for WHtR in both sex, BRI 3.58 in males and 3.62 in females) [47]. In another study, the cut-off points of the WC, WHtR, and BRI indices for predicting metabolic syndrome (MetS) were reported 87.25, 0.51, and 3.55 in males and 77.25, 0.49, and 3.18 in females, respectively [23].

The ability of AVI, BRI, and BAI was assessed as a predictor factor of metabolic syndrome and cardiovascular diseases in several studies [48, 49]. Current study showed the BRI, WHR, WHtR, and AVI indices strongly predict the risk of malnutrition among both sexes. The odds ratio (OR) was closely for other anthropometric indices. Yang et al. showed that the OR of prediction of diabetes among older persons for BRI was greater than other anthropometric indices in both sexes [47]. Hu et al. showed that BMI, WC, and WHtR were independently associated with all-cause mortality among older persons [28]. The high prevalence of malnutrition among older persons in Turkey [19] necessitates anthropometric assessment of the older persons and this relatively can prevent and reduce the dangers of malnutrition.

In summary, although measuring BMI as common index for assessing malnutrition, the other anthropometric indices should be considered. In other words, BMI in combination with other anthropometric indices can better reflect health status.

The main strength of this study is the large sample size, and the population screening for the assessment of malnutrition status of older people. To increase accuracy, the anthropometric measurements were taken from each participants by two staffs. The limitation of this study is that the health status and illnesses among subjects were not studied.

## Conclusion

Current study showed that there were differences among males and females in the cutoffs of HC, WC, WHR, WHtR, AC, MUAC, TST, CC, AVI, BRI, and BAI. Because the AUC was > 0.7 in all anthropometric measurements then these indices could be utilized to assess risk of malnutrition among older people in both sexes.

## Data Availability

The datasets that support the findings of this study are available on request from the first author [Özgün Başıbüyük, G.]. (As the row data are still under process of evaluation, analysis, and further publication the data are not publicly available).

## Abbreviations

HC: Hip circumference
WC: Waist circumference
AC: Abdominal circumference
MUAC: Mid-upper arm circumference
TST: Triceps skinfold thickness
CC: Calf circumference
BMI: Body mass index
AVI: Abdominal volume index
BRI: Body roundness index
BAI: Body adiposity index
WHR: Waist-to-hip ratio
WHtR: Waist-to-height ratio
ROCs: Receiver operator characteristic curves
OR: Odds ratios
AUC: Area under the curve
